# Launching “methods and statistics tutorials”: A collection of resources for systematic reviewers

**DOI:** 10.1002/cesm.12017

**Published:** 2023-06-18

**Authors:** Kerry Dwan, Rachel Richardson

**Affiliations:** ^1^ Centre for Evidence Synthesis in Global Health Liverpool School of Tropical Medicine Liverpool UK; ^2^ Methods Support Unit Cochrane London UK

1

Robust and innovative methods are the lynchpin of Cochrane and remain the basis of its reputation as the home of high‐quality systematic reviews. As methodology evolves to respond to the evidence needs of our stakeholders, it is crucial for Cochrane to be able to offer timely and clear advice to those working on our reviews.

Cochrane's Methods Support Unit (MSU) was established in 2019 [1] to provide methodological and statistical support to authors and editors working on Cochrane protocols and reviews. A key challenge in the first 2 years of the Unit was the introduction of our new tool [2] to assess the risk of bias in randomized controlled trials (RCTs), but we have dealt with a multiplicity of different issues since being established. By March 2023, the Unit has dealt with over 1200 referrals relating to protocols, systematic reviews, updates of reviews, and statistical and methodological queries relating to reviews. Our work has ranged from full methodological and statistical reviews of research incorporating network meta‐analyses to individual queries about the use of an intracluster correlation coefficient to adjust data from an individual study.

It quickly became apparent that similar queries were often raised and we noticed common problems when working on reviews. Our monthly web clinic series was set up to allow authors and editors to raise questions and then later to provide guidance on a specific topic in addition [3]. However, we also felt that there is a need for accessible help with these issues which would be instantly available.

Professor Doug Altman was a leader and pioneer in Cochrane and in the field of medical statistics [4]. One of Professor Altman's legacies was the Statistics Notes series in *The BMJ*, which provided clear advice on statistical concepts to medical researchers. Inspired by this work, former and current MSU managers (Dr Kerry Dwan and Rachel Richardson) are launching this series to provide easily accessible advice to the evidence synthesis community on the common methodological and statistical issues that we have observed within Cochrane. The hope is to make this as interactive and easy to understand as possible. In addition, the series will collaborate with Cochrane Training [5] to complement the articles in the series with visual learning, such as short videos or quick e‐learning checks, to help those who learn better by doing or watching rather than reading.

There are various sources of advice on methodological and statistical issues already available to reviewers, including the Cochrane Handbook for Systematic Reviews of Intervention [6] and resources from specialized Cochrane Methods Groups. This series will complement existing sources by responding to the day‐to‐day difficulties encountered by reviewers when implementing systematic review methods. The MSU is in a unique position to provide advice; having dealt with thousands of queries and requests for peer review since our inception, we know what reviewers find difficult and also why these issues cause confusion. We also believe that linking the articles with resources from Cochrane Training will make this series uniquely useful.

Examples of common issues authors face when working on systematic reviews include:

- dealing with data from crossover RCTs;
- the correct way to undertake and interpret subgroup analyses;
- inclusion of multiarm trials in meta‐analysis;
- heterogeneity;
- the use of fixed versus random effects models in meta‐analysis.

Early articles in the series will focus on the use of effect measures in systematic reviews, specifically the standardized mean difference and the use of risk ratios versus odds ratios. These two topics often cause difficulties for authors and editors. Examples will be given in the articles of when to use these measures, how to calculate them and their interpretation. We will also cover cluster RCTs early in the series.

We welcome proposals for this series from members across the evidence synthesis community on areas they see as methodological and statistical challenges for authors. Articles should be short tutorials, use the Commentary article type, and, in general, be less than 1000 words.

Cochrane's mission is to produce trusted synthesized evidence, make it accessible to all, and advocate for its use. Ensuring that evidence synthesis methods are implemented wisely and accurately is foundational to us being able to achieve this mission. With the Methods and Statistics Tutorials series, we hope to support and onboard a wider and more diverse community of authors to produce the next generation of high‐quality Cochrane Reviews and other evidence syntheses.

## AUTHOR CONTRIBUTIONS

**Kerry Dwan**: Conceptualization; writing—original draft; writing—review and editing. **Rachel Richardson**: Conceptualization; writing—review and editing.

## FUNDING INFORMATION

None

## PEER REVIEW

The peer review history for this article is available at https://www.webofscience.com/api/gateway/wos/peer-review/10.1002/cesm.12017.

## Data Availability

Data sharing is not applicable to this article as no new data were created or analyzed in this study.

## References

[1] Dwan K . Launching the new methods support unit in Cochrane. Cochrane Methods, Cochrane News. Accessed April 2023. https://methods.cochrane.org/news/launching-new-methods-support-unit-cochrane

[2] Risk of bias 2 (RoB 2) tool. Cochrane Methods . Accessed April 2023. https://methods.cochrane.org/risk-bias-2

[3] Methods support unit web clinics. Cochrane Training . Accessed April 2023. https://training.cochrane.org/learning-events/learning-live/methods/msu-web-clinics

[4] In memoriam: Doug Altman. Cochrane Community . Accessed April 2023. https://community.cochrane.org/news/memoriam-doug-altman

[5] Cochrane Training. Trusted evidence. Informed Decisions. Better Health . Accessed April 2023. https://training.cochrane.org/

[6] Cochrane handbook for systematic reviews of interventions. Cochrane Training . Accessed May 2023. https://training.cochrane.org/handbook

